# The unstructured domain of colicin N kills *Escherichia coli*

**DOI:** 10.1111/mmi.12260

**Published:** 2013-06-05

**Authors:** Christopher L Johnson, Helen Ridley, Robert J Pengelly, Mohd Zulkifli Salleh, Jeremy H Lakey

**Affiliations:** Centre for Bacterial Cell Biology, Institute for Cell and Molecular Biosciences, Faculty of Medical Sciences, Newcastle UniversityFramlington Place, Newcastle- upon-Tyne, NE2 4HH, UK

## Abstract

Bacteria often produce toxins which kill competing bacteria. Colicins, produced by and toxic to *Escherichia coli* bacteria are three-domain proteins so efficient that one molecule can kill a cell. The C-terminal domain carries the lethal activity and the central domain is required for surface receptor binding. The N-terminal domain, required for translocation across the outer membrane, is always intrinsically unstructured. It has always been assumed therefore that the C-terminal cytotoxic domain is required for the bactericidal activity. Here we report the unexpected finding that in isolation, the 90-residue unstructured N-terminal domain of colicin N is cytotoxic. Furthermore it causes ion leakage from cells but, unlike known antimicrobial peptides (AMPs) with this property, shows no membrane binding behaviour. Finally, its activity remains strictly dependent upon the same receptor proteins (OmpF and TolA) used by full-length colicin N. This mechanism of rapid membrane disruption, via receptor mediated binding of a soluble peptide, may reveal a new target for the development of highly specific antibacterials.

## Introduction

For many years the structure–function paradigm governed our view of protein biochemistry until in the last decade it became clear that intrinsically unstructured domains (IUDs) are critical to many processes. IUDs are most commonly involved in protein–protein interactions; however, they are also involved in protein–DNA and protein–RNA binding. Their inherent disorder is fundamental to their biological roles. The intrinsic flexibility of IUDs confers several functional advantages, such as specificity without excessive binding strength, increased speed of interaction and binding promiscuity making IUDs ideal for signalling and regulatory processes (Meszaros *et al*., 2011). Thus they are implicated in transcription, translation and cell signalling in all three domains of life and conservative estimates indicate at least 25% of sequences in SwissProt contain long disordered regions (Romero *et al*., 2001). One of the best-studied examples of a protein with IUDs, the crucial tumour suppressor protein p53, exhibits a highly biased use of disordered regions for mediating and modulating interactions with other proteins, utilizing different regions in the disordered tails to enable binding to multiple partners' simultaneously (Oldfield *et al*., 2008). Here we add antibacterial activity to their list of roles and interestingly this newly discovered function also relies upon the ability to bind two different proteins.

The appearance of antibiotic-resistant and novel pathogenic strains of *Escherichia coli* (*E. coli*) emphasizes the continuing need for new antibacterial treatments. AMPs are produced by a wide variety of living organisms from bacteria to animals and are a potentially large antibiotic resource (Melo *et al*., 2009). They often kill bacteria by membrane disruption which is a highly effective and rapid mechanism. However, since human and bacterial membranes are similar; it is often difficult to target only the bacteria and avoid damage to the patient's cells. Thus in order to viably use membrane disrupting molecules in antibacterial chemotherapy their specificity needs to be improved.

Bacteria often compete among themselves for resources and this selection pressure has encouraged the widespread evolution of antibacterial behaviour in bacteria. Often this involves the secretion of antibacterial molecules into the shared extracellular space leading to inhibition or death of the competitor cells. These antibacterial proteins are collectively called bacteriocins and include peptides (e.g. nisin), small proteins (e.g. pediocins) and larger proteins such as the pyocins (*Pseudomonas*) and pesticins (*Yersinia pestis*). Unlike traditional antibiotics, bacteriocins are often only toxic to bacteria closely related to the producing strain (Riley and Wertz, 2002).

*Escherichia coli* produces two classes of bacteriocins (Gordon and O'Brien, 2006) which are typically plasmid encoded. These are the low-molecular-weight microcins and the larger (> 30 kDa) colicins. Microcins range from 1 to 10 kDa and have minimum inhibitory concentrations (MICs) in the nanomolar range. They disrupt a wide range of functions in the target cell including ATP synthetase (Rodriguez and Lavina, 2003) and DNA gyrase (Heddle *et al*., 2001). In general, translocation of microcins into the target cell requires proteins of the Ton system (Duquesne *et al*., 2007) which normally import low abundance nutrients. The Ton system comprises an outer membrane receptor (e.g. Cir), the periplasm-spanning TonB protein and two inner membrane proteins, ExbB and ExbD. Some microcins imitate the structures of nutrients and this may define their uptake pathway (Strahsburger *et al*., 2005).

Colicins range in size from 30 to 80 kDa and are extremely efficient toxins with MICs in the pM–nM range; requiring only one molecule to kill a target cell. They play an important role in gut colonization by new strains and have varied killing mechanisms which include pore-formation, DNase or RNase activity or inhibition of peptidoglycan biosynthesis (Cascales *et al*., 2007). Some colicins (Group B) exploit the Ton system but others (Group A) hijack the Tol protein system (TolA, TolB, TolQ and TolR) (Cascales *et al*., 2007) which is unexploited by microcins. TolA resembles TonB, spans the periplasm and is coupled to the proton motive force but, unlike TonB, is not linked to nutrient transport. Rather, TolA is implicated in outer membrane stability and cell division (Gerding *et al*., 2007).

Regardless of the mechanism of import, all colicins have the same modular three-domain structure; an N-terminal translocation (T) domain, a central receptor binding (R) domain and a C-terminal cytotoxic domain which carries the lethal activity (Braun *et al*., 2002). In every colicin the N-terminal T-domain contains an IUD with no defined 3D structure. These IUDs contain the TolA, TolB and TonB binding regions and join a growing list of IUDs which have been shown to play key roles in molecular recognition, cell regulation and disease in all proteomes (Dunker and Kriwacki, 2011). C-terminal domains vary in structure according to function and it is possible to exchange the toxic activity of a colicin (e.g. pore-former to DNase) by swapping the C-terminal domain for another type (Benedetti *et al*., 1991). Thus while the C-terminus contains and determines the toxic function, the remaining two domains are traditionally viewed as the vehicle which efficiently transports the toxicity into the cell.

Most colicins use two different types of outer membrane receptors, e.g. in ‘E’ colicins the high-affinity receptor, BtuB, is recognized by the tip of the coiled-coil R-domain (Penfold *et al*., 2004; Sharma *et al*., 2007). The secondary receptor, or translocator, OmpF is then recruited by the IUD T-domain (Housden *et al*., 2005; 2011). Other colicins require only one type of outer membrane receptor and here the best-studied example is ColIa (Buchanan *et al*., 2007; Udho *et al*., 2009). Crucially for the work reported here it has recently been demonstrated that ColIa requires two copies of its receptor Cir; one for reception and one for translocation (Jakes and Finkelstein, 2010).

Colicin N (ColN), which we study, is a colicin which requires a single type of outer membrane protein, OmpF. As with all colicins it has always been assumed that the sole role of the ColN T-domain (Pugsley, 1987; Bourdineaud *et al*., 1990; Fourel *et al*., 1990; El Kouhen *et al*., 1993; El Kouhen and Pages, 1996) was to deliver the cytotoxic C-terminal domain across the outer membrane (Kleanthous, 2010). Here we report the unexpected finding that the isolated unstructured T-domain of ColN kills *E. coli* in an OmpF and TolAQR-dependent manner. This is the first example of bactericidal membrane disruption by an IUD. Furthermore, we also show that (like ColIa) ColN hijacks two copies of its outer membrane receptor during translocation.

## Results

### The cytotoxic C-terminal pore-forming domain of ColN is dispensable for antimicrobial activity

**Fig. 1:**
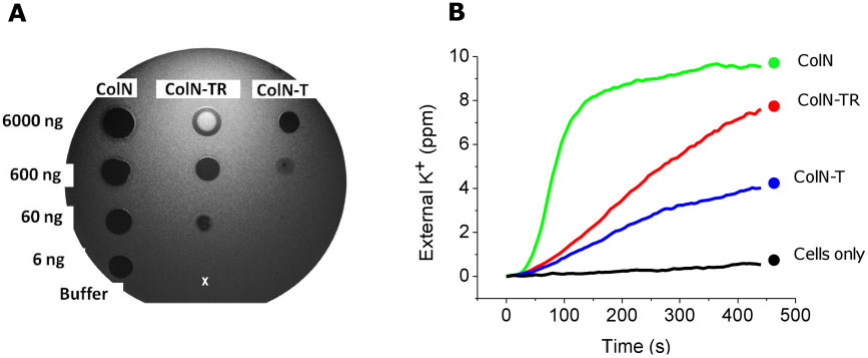
Comparing of ColN, ColN-TR and ColN-T activity. A. Spot test assay to demonstrate killing of *E. coli* cells by colicin constructs which lack the P-domain. Two microlitre volumes corresponding to 6000 ng, 600 ng, 60 ng and 6 ng of ColN, ColN-TR and ColN-T were spotted onto a lawn of *E. coli*. As a negative control 2 μl of the buffer in which the colicins were diluted (50 mM sodium phosphate, pH 7.5, 300 mM NaCl) was spotted (x). B. K^+^ efflux assay to compare the activity of ColN added at 600 molecules per cell (mpc) (green), ColN-TR added at 10^5^ mpc (red), ColN-T added at 2 × 10^5^ mpc (blue) compared with a negative control to which no colicin is added to cells (black).

When using a simple spot test assay to screen ColN deletion mutants, we found that the pore-forming domain of ColN (ColN-P) was dispensable for antimicrobial activity. Both the T- and R-domains combined (ColN-TR) and the T-domain in isolation (ColN-T) produced zones of clearing (Fig. 1A) when applied to a lawn of *E. coli*, the same feature observed with ColN (e.g. ColN-TRP). ColN showed killing activity when as little as 6 ng of protein was added to the lawn of *E. coli*, ColN-TR needed 60 ng and ColN-T 600 ng. Additionally, we observed a halo when spotting ColN-TR at its highest concentration (6000 ng). The halo represents live cells in the centre of the spot and a zone of clearing at the periphery. This suggests ColN-TR displays self-inhibition of killing in the centre of the spot where the concentration of ColN-TR is at its highest, a phenomenon also seen with ColIa (Jakes and Finkelstein, 2010).

### ColN-TR and ColN-T are potent antimicrobials which display unique specificity

**Fig. 2:**
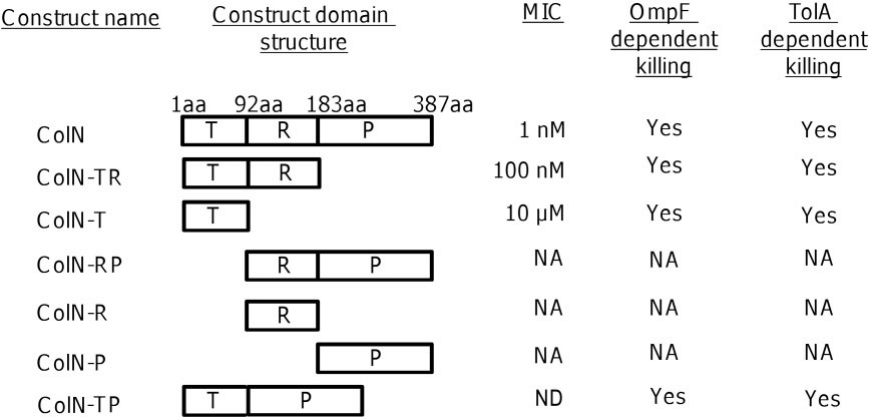
Schematic of ColN domain mutants used in this work and their associated minimum inhibitory concentrations (MICs) and OmpF/TolA dependencies. NA: not active in the range 1 pM–10 μM. ND: not determined.

We next addressed the cytotoxic activity of ColN-TR and ColN-T. K^+^ efflux assays are an extremely sensitive quantitative assay of ColN activity, measuring the real-time release of intracellular K^+^ from *E. coli*. These revealed that both ColN-TR and ColN-T provoked K^+^ efflux from cells, albeit less efficiently than ColN (Fig. 1B). This trend was repeated in the MICs (Fig. 2). ColN was found to have a MIC of 1 nM, a result anticipated from the literature (Sharma *et al*., 2009). ColN-TR and ColN-T were found to have MICs of 100 nM and 10 μM respectively which are comparable to known AMPs (Melo *et al*., 2009). To test the specificity of this newly discovered antimicrobial activity, spot test assays against *E. coli* cells lacking either OmpF or TolA were carried out. In each case, ColN-TR and ColN-T are non-toxic (Fig. S1A and B) and thus, like full-length ColN, they are strictly dependent upon both OmpF and TolA. ColIa was used as a positive control since it relies upon Cir and TonB and therefore has no requirement for OmpF or TolA. ColIa not only produced zones of clearing in the absence of either OmpF or TolA, but also showed the previously described halo of self-inhibition (Jakes and Finkelstein, 2010). ColN binds TolA directly but its activity also depends upon the whole inner membrane complex TolAQR (Sharma *et al*., 2009). The susceptibility of Δ*tolQ* (*tolQ786::kan*) and Δ*tolR* (*tolR787::kan*) strains to ColN, ColN-TR and ColN-T was tested, alongside the wild-type Keio strain BW25113 [*rrnB3* Δ*lacZ4787 hsdR514* Δ(*araBAD*)*567* Δ(*rhaBAD*)*568*] (Baba *et al*., 2006) (Fig. S1C–E). BW25113 showed susceptibility to all three constructs as would be expected for a wild-type strain (Fig. S1A). Both Δ*tolQ* and Δ*tolR* strains showed expected partial resistance to ColN but complete resistance to both ColN-TR and ColN-T (Fig. S1D and E). This demonstrates that the entire TolAQR inner membrane complex is essential for killing by ColN-TR or ColN-T. TolB on the other hand is not required for toxicity by ColN, ColN-T or ColN-TR (results not shown).

### Isolation of an OmpF binding site in ColN-T

The OmpF dependence of ColN-T correlates with the recent demonstration of two OmpF binding sites within ColE9-T (Housden *et al*., 2011). Short regions of predicted order bounded by extended regions of predicted disorder (predicted using PONDR VL-XT) have been shown in several cases to identify binding sites that undergo disorder-to-order transitions upon complex formation (Garner *et al*., 1998), with the disorder-to-helix transition being particularly common (Fuxreiter *et al*., 2004). PONDR analysis (Romero *et al*., 1997; 2001; Garner *et al*., 1998; Li *et al*., 1999; Fuxreiter *et al*., 2004) predicted an ordered region within the IUD spanning residues 8–15 (Hecht *et al*., 2009). This region was tentatively termed OBS1 [OmpF binding site 1 (Housden *et al*., 2011)]

Next we carried out multiple sequence alignments of ColN against other colicins which use OmpF for import (Fig. S2A and B). Alignment of ColN-T with the OmpF-dependent pore-forming colicins; colicin K (ColK) and colicin A (ColA) reveals low homology in the ColN OBS1 region with only loose conservation of glycine residues, as expected in such glycine-rich IUDs. We next aligned ColN with the known OBS1 and 2 sites of ColE9. This reveals little similarity to either OBS1 or OBS2 regions; however, the di-peptide sequence of (His)-(Asn) is conserved in ColE9, ColE3 and ColN. PONDR analysis of the colicins used in the multiple alignments (Fig. S3A) revealed that the N-terminal ∼ 10 aa of colicin A (ColA) are predicted to form an ordered region which encompasses the region that aligns with the ColN OBS1 site (Fig. S2A). PONDR analysis of the enzymatic colicins ColE9 and ColE3 predicted no ordered regions coincidental with OBS1 and OBS2 sites of ColE9 (Fig. S3).

**Fig. 3:**
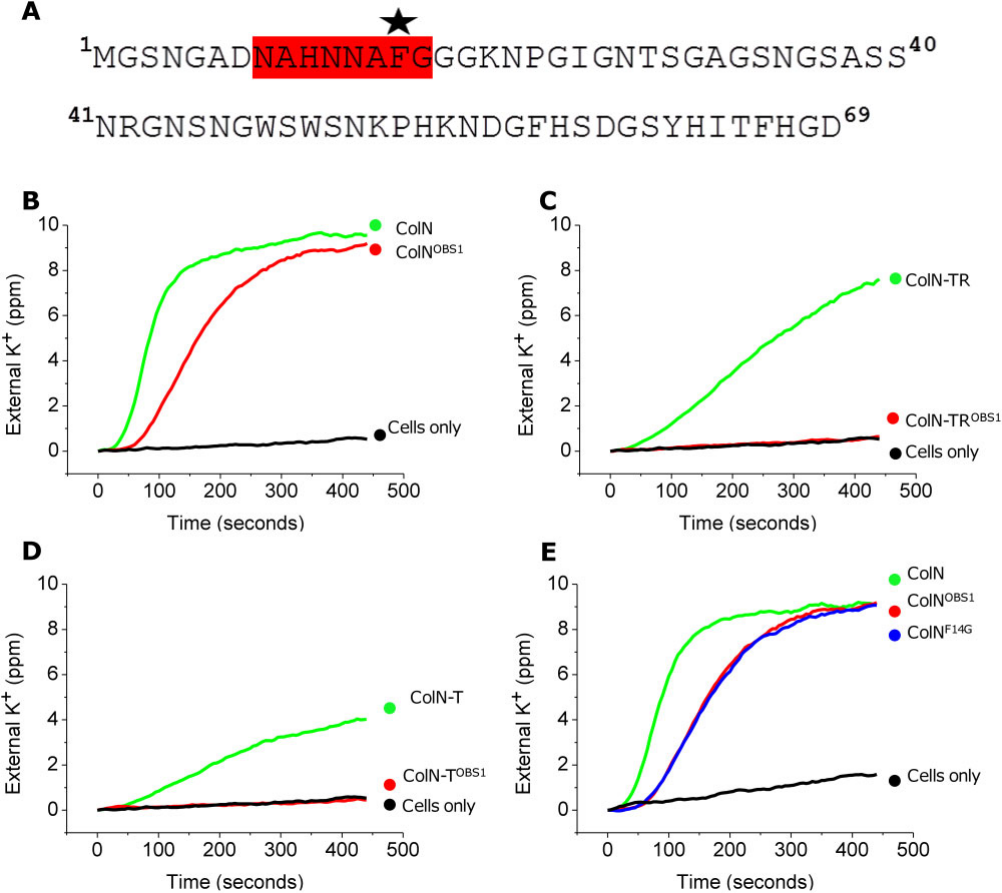
K^+^ efflux assays of OBS1 mutants in ColN, ColN-TR and ColN-T. A. Sequence of the first 69 amino acids of ColN highlighting the OBS1 mutation (red box) and F14 (black star); NB: the complete OBS1 site is yet to be defined. B. ColN (green) and ColN^OBS^^1^ (red) added at 600 mpc, negative control (black). C. ColN-TR (green) and ColN-TR^OBS^^1^ (red) added at 10^5^ mpc, negative control (black). D. ColN-T (green) and ColN-T^O^^BS^^1^ (red) added at 2 × 10^5^ mpc, negative control (black). E. A direct comparison of the K^+^ efflux induced by ColN^OBS^^1^ (red) and ColN^F^^14G^ (blue) added at 600 mpc. ColN (green) added at 600 mpc is shown for reference, negative control (black).

To experimentally test the role of OBS1 in OmpF binding we mutated the region (N^8^AHNNAFG^15^ to N^8^GGSGGS G^15^) (Fig. 3A) to generate ColN^OBS1^, ColN-TR^OBS1^ and ColN-T^OBS1^ respectively. ColN^OBS1^ provoked K^+^ efflux which was only modestly slower than ColN (Fig. 3B). However ColN-TR^OBS1^ and ColN-T^OBS1^ were completely inactive (Fig. 3C and D). Histidine 10 (H10) and phenylalanine 14 (F14) contribute the only large amino acid side-chains in OBS1 so we made the point mutants ColN^H10G^ and ColN^F14G^. ColN^H10G^ was less active than ColN but more active than ColN^OBS1^ (Fig. S4A) while ColN^F14G^ displayed activity which overlaid with ColN^OBS1^ (Fig. 3E), demonstrating that F14 is a critical residue in OBS1. We also assessed the activity of ColN missing the N-terminal 40 residues ColN^(41–387)^ (Fig. S4B) which removes OBS1 but leaves the TolA binding site intact. The K^+^ efflux of ColN^(41–387)^ was found to be more rapid than ColN^OBS1^ (Fig. S4B). This small increase may be due to the residual N-terminus of ColN^OBS1^ forming unproductive interactions with OmpF. We next used spot test assays and MIC tests. Spot test assays failed to detect any difference in activity between ColN^OBS1^, ColN^F14G^ and ColN^H10G^ (Fig. S5), while the more sensitive MIC test did detect differences in their activities (Table S1). The MIC value obtained for ColN^OBS1^ and ColN^F14G^ was 10 nM while that of ColN^H10G^ was found to be 1 nM, the same as ColN. In summary, this newly identified OmpF binding site within ColN-T is essential for the toxicity of both ColN-TR and ColN-T; however, its loss can be tolerated in full-length ColN with only a modest reduction in activity. Since both the OBS1 site and the TolA interaction are essential for toxicity we produced a colicin construct consisting of residues 1–69 with a C-terminal His tag (ColN-T^(1–69)^) encompassing OmpF and TolA binding boxes and removing residues 70–90 to which no function has yet been assigned. The activity of ColN-T^(1–69)^ was compared with ColN-T (i.e. 1–90) by K^+^ efflux. ColN-T^(1–69)^ provoked very similar K^+^ efflux to ColN-T (Fig. S4D).

### OBS1 is essential for OmpF binding *in vitro*

Next, we wished to show that the loss of K^+^ efflux activity in ColN-TR^OBS1^ and ColN-T^OBS1^ was due to disruption of OmpF binding. To do this we probed the binding of ColN, ColN-TR, ColN-T, ColN^OBS1^, ColN-TR^OBS1^ and ColN^F14G^ to OmpF using isothermal titration calorimetry (ITC). ColN, ColN-TR and ColN-T all bound to OmpF with similar affinities, demonstrating that it is ColN-T which drives OmpF binding under these conditions (Table 1). The thermodynamic parameters (negative *TΔS*) are consistent with the disordered T-domain becoming ordered upon complex formation.

**Table 1:** Thermodynamic analysis of the binding of colicin mutants to OmpF

	*Kd*, μM	N	*ΔH*, kcal mol^−1^	*TΔS*, kcal mol^−1^	*ΔG*, kcal mol^−1^	Source
ColN	2.10 ± 0.70	2.40 ± 0.20	−12.31 ± 2.01	−4.54 ± 2.13	−7.74 ± 0.17	Evans *et al*. (1996a)
ColN-TR	1.60 ± 0.18	2.76 ± 0.01	−17.29 ± 1.58	−9.37 ± 1.65	−7.92 ± 0.07	This study
ColN-T	3.48 ± 0.03	2.29 ± 0.03	−19.09 ± 0.37	−11.62 ± 0.39	−7.46 ± 0.02	This study
ColN^OBS1^	NB	NB	NB	NB	NB	This study
ColN-TR^OBS1^	NB	NB	NB	NB	NB	This study
ColN^F14G^	NB	NB	NB	NB	NB	This study

ITC parameters for the binding of ColN, ColN-TR, ColN-T, ColN^OBS1^ ColN-TR^OBS1^ and ColN^F14G^ to OmpF trimers at 25°C in 20 mM potassium phosphate, pH 7.5, 150 mM NaCl, 1% Octyl-poe. The errors shown are those from duplicate experiments. NB: no binding (heats of interaction are no greater than heats of dilution).

Remarkably, the OBS1 mutation in ColN^OBS1^, ColN-TR^OBS1^ completely abolished OmpF binding. Presumably OmpF binding would also be abolished in ColN-T^OBS1^; however, since ColN-T^OBS1^ is unstable and we could not produce sufficient quantities for ITC, this experiment was not carried out. Finally we probed the interaction of the single point mutant ColN^F14G^ with OmpF. As with the longer OBS1 mutation, this single point mutation completely abolished OmpF binding. In summary these results demonstrate the OmpF binding site in ColN-T overlaps OBS1 and further support the role of F14 as a critical residue in OBS1.

### ColN^OBS^^1^ mutant activity is easily blocked by non-functional ColN^Δ^^TolA^ mutants

**Fig. 4:**
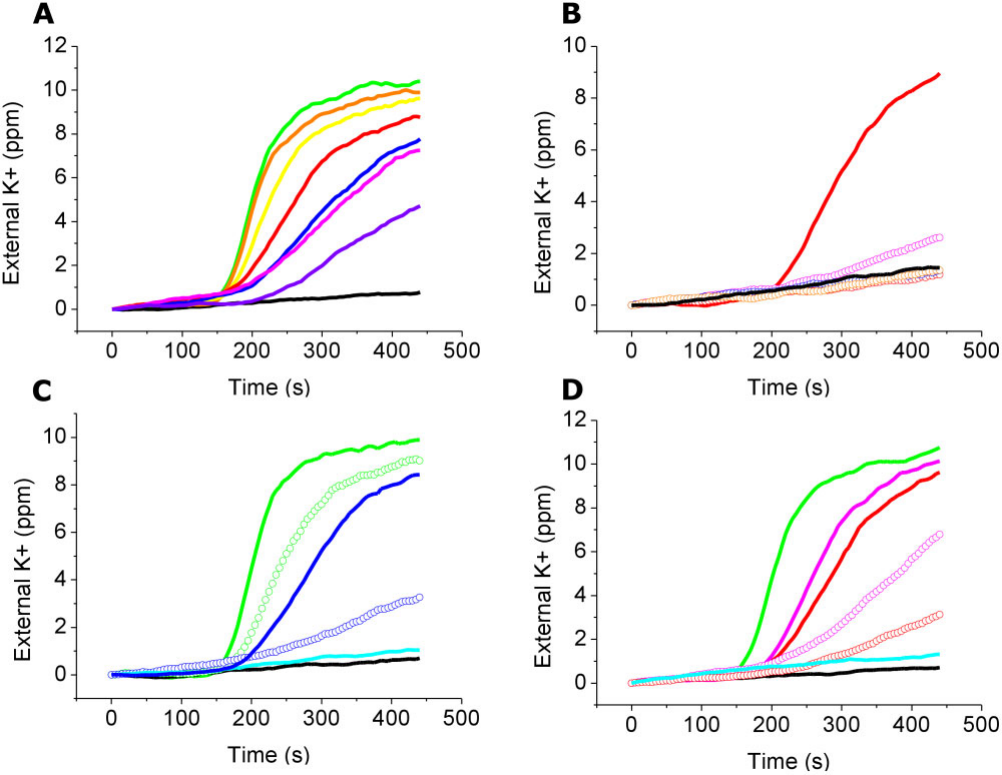
K^+^ efflux assays to assess the blocking of OBS1 mutants by ColN^ΔTolA^ (A and B) and ColN-RP (C and D). A. ColN (green) was added at 600 mpc at 120 s as an unblocked control. For all other blocking runs ColN^ΔTolA^ was added after 60 s at increasing multiplicities (orange = 10^4^, yellow = 10^5^, red = 1.3 × 10^5^, blue = 1.6 × 10^5^, magenta = 2 × 10^5^, purple = 3 × 10^5^ mpc) followed by ColN at 600 mpc at 120 s. Negative control of ColN^ΔTolA^ added at 2 × 10^5^ mpc (black). B. ColN^OBS^^1^ (solid red line) was added at 600 mpc at 120 s as an unblocked control. For all other runs ColN^ΔTolA^ was added at 2 × 10^5^ mpc at 60 s followed by either ColN^OBS^^1^ (red open circles), ColN^F^^14^^G^ (open blue circles), ColN^H^^10^^G^ (open orange circles) or ColN(^41–387)^ (open magenta circles) at 600 mpc at 120 s. Negative control to which no colicin is added to cells (black). C. ColN (solid green line) and ColN^F^^14^^G^ (solid blue line) were added at 600 mpc at 120 s as unblocked controls. For all other blocking runs ColN-RP was added at 2 × 10^5^ mpc at 0 s followed by ColN (green open circles) or ColN^F^^14G^ (blue open circles) at 600 mpc at 120 s. Negative control to which no colicin is added to cells (black solid line). ColN-RP added at 2 × 10^5^ mpc at 0 s (solid cyan line). D. ColN (solid green line), ColN^OBS^^1^ (solid red line) and ColN^(41–387)^ (solid magenta line) were added at 600 mpc at 120 s as unblocked controls. For all other blocking runs ColN-RP was added at 2 × 10^5^ mpc at 0 s followed by either ColN^OBS^^1^ (red open circles) or ColN^(41–387)^ (magenta open circles) at 120 s. Negative control to which no colicin is added to cells (black solid line), ColN-RP added at 2 × 10^5^ mpc at 0 s (solid cyan line).

ColN^ΔTolA^ (lacking residues 40–69) which retains functional OBS1 site but no TolA (periplasmic receptor) binding ability appears to form a stalled complex in the colicin translocon. Experiments revealed that it partially blocks the activity of ColN; when added at 3 × 10^5^ molecules per cell (mpc) it brought about a ∼ 50% reduction in K^+^ efflux (Fig. 4A). The requirement for high multiplicities implies that ColN^ΔTolA^ is inhibiting ColN at the OmpF binding stage (∼ 10^5^ OmpF molecules per cell). Significantly, although ColN^OBS1^ and ColN^F14G^ were completely blocked by the same amount of ColN^ΔTolA^, ColN^(41–387)^ could partially overcome ColN^ΔTolA^ blocking (Fig. 4B), but not as effectively as full-length ColN. ColN-RP blocked ColN activity much less than ColN^ΔTolA^; however, this ‘ΔOBS1 + ΔTolA’ mutant still blocked significantly against ColN^F14G^ (Fig. 4C) and ColN^OBS1^ (Fig. 4D). Finally ColN^(41–387)^ overcame ColN-RP blocking significantly better than proteins with simple mutations in the OBS1 site (Fig. 4D). Thus ColN^ΔTolA^ and ColN-RP fully block the action of ColN OBS1 mutants but cannot entirely block full-length ColN or ColN^(41–387)^.

### ColN-T shows no evidence of membrane interactions

**Fig. 5:**
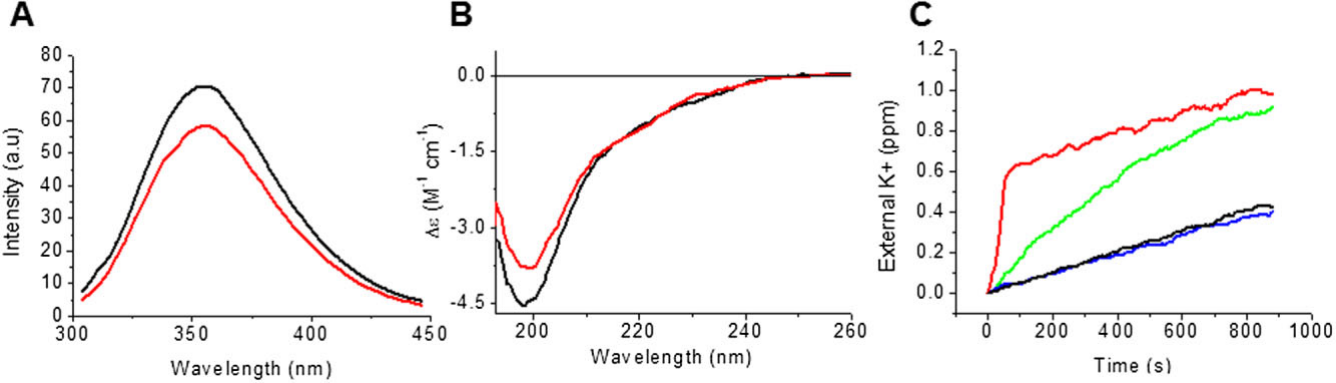
ColN-T shows no evidence of interaction with model membranes. A. Tryptophan fluorescence. Buffer (20 mM Tris-HCl, pH 7.5, 300 mM NaCl) (black) or *E. coli* lipid liposomes composed of natural *E. coli* phospholipids (red) were mixed with ColN-T (25 μg ml^−1^) and fluorescence emission spectra recorded from 300 to 450 nm. B. Circular dichroism analysis to compare the secondary structure of ColN-T in 10 mM sodium phosphate pH 7.5, 150 mM NaCl (black) and 10 mM NaP pH 7.5, 150 mM NaCl, 1 mM SDS (red). C. K^+^ efflux assay to determine if ColN-T can cause K^+^ leakage from K^+^ loaded liposomes. K^+^ loaded liposomes were added at 0 s followed by ColN-T at 1 μM (blue), melittin at 1 μM (green) or 10 μM (red) at 60 s. To assess the basal leakage from K^+^ loaded liposomes a negative control was carried out where nothing was added to the liposomes (black).

Since ColN-T provoked K^+^ leakage from cells, it appeared likely that ColN-T was acting like pore-forming AMPs, of which many have been described (Brogden, 2005). AMPs can be unfolded in solution but fold on contact with lipid membranes, disrupting the inner membrane by the formation of structured pores, although some may go on to attack other intracellular targets. To discover whether ColN-T was an AMP able to disrupt the inner membrane, we assessed its interaction with model membranes. Fluorescence experiments (Fig. 5A) showed no shift in the ColN-T tryptophan spectrum in the presence of *E. coli* lipid liposomes demonstrating that ColN-T does not readily interact with *E. coli* phospholipids. In the presence of SDS, circular dichroism experiments (Fig. 5B) showed no formation of alpha helix structure, characteristic of many pore-forming peptides. This suggests that ColN-T does not behave like other AMPs and remains as an unstructured peptide in the presence of membranes. We next prepared natural *E. coli* lipid liposomes filled with KCl and assessed the ability of ColN-T to cause K^+^ leakage. Under these conditions the loss of K^+^ from the liposomes when ColN-T was added at 1 μM was the same as the negative control of liposomes to which nothing was added. However, melittin, used as a positive control provoked significant K^+^ leakage at both 1 μM and 10 μM (Fig. 5C). ColN-T thus requires additional factors to disrupt the membrane such as protein receptors and/or an energized membrane.

### ColN-TR displays self-inhibition at high concentrations

**Fig. 6:**
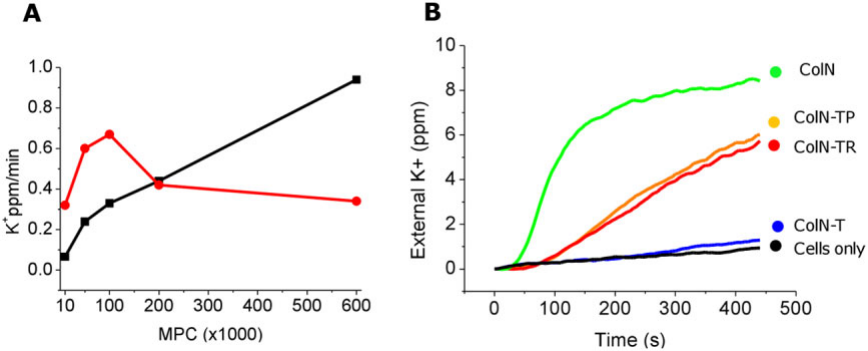
Comparing ColN-TR and ColN-TP activities. A. Self-inhibition of killing using ColN-TR. A comparison of the rate of K^+^ efflux induced by ColN-T and ColN-TR when added at 10^4^, 5 × 10^4^, 10^5^, 2 × 10^5^ and 6 × 10^5^ mpc. The rate of efflux was calculated from the linear region of the efflux curve. The rate of efflux induced by ColN-T increases with multiplicity while ColN-TR has a maximal rate when added at 10^5^ mpc. B. K^+^ efflux induced by ColN-TP, ColN-TR and ColN-T at 2.5 × 10^4^ mpc. ColN-TP (orange line), ColN-TR (red line) and ColN-T (blue line) were added at 2.5 × 10^4^ mpc at 0 s. ColN (green) added at 600 mpc at 0 s is shown for reference. Negative control to which no colicin is added to cells (black solid line).

ColN-TR self-inhibition at high concentrations, as exhibited on spot test assays (Fig. 1A), was further investigated using the more quantitative K^+^ efflux assay. ColN-TR and ColN-T were added to *E. coli* cells at levels ranging from 10^4^ to 6 × 10^5^ molecules per cell (mpc) (Fig. 6A). The rate of K^+^ efflux induced by ColN-T increased steadily with concentration but below 10^5^ mpc ColN-TR is a more efficient killer than ColN-T, the rate of K^+^ efflux being at least double that of ColN-T. However ColN-TR, showed a distinct optimum at 10^5^ mpc after which the rate decreased such that 6 × 10^5^ mpc provided approximately the same rate of efflux as 10^4^ mpc. This decrease in rate suggests that the R-domain is causing self-inhibition of T-domain killing.

Removal of the R-domain (ColN-TP) also results in a toxic protein. K^+^ efflux experiments revealed that ColN-TP provoked K^+^ efflux at a similar rate to ColN-TR (non-self-inhibiting multiplicity) of 2.5 × 10^4^ mpc (Fig. 6B). ColN-TP did not show self-inhibition (results not shown) indicating a more specific role of the R-domain in OmpF binding. Thus either the P-domain in TP is assisting the docking of T-domain to the OmpF or, if it is forming pores in the cytoplasmic membrane, its activity is very much reduced.

### Post-translational modification of ColN-T under prolonged induction conditions

To increase the yield of ColN-T purification (typically 0.5 mg l^−1^) induction times were increased from 2 h to 16 h. Surprisingly, although yields of pure protein increased significantly, the antibacterial activity was often low or zero and these samples did not behave as a protein with a pI of 9.3. In cation exchange chromatography using 20 mM potassium phosphate, pH 7.5, 150 mM NaCl as the loading buffer, ∼ 90% of ColN-T loaded onto the column did not bind. This fraction was inactive whereas the ColN-T which bound and was then eluted from the column as normal behaved as ColN-T produced under 2 h induction conditions.

Given the structure of ColN-T, the most likely cause is the deamidation of asparagine residues in Asn–Gly pairs, producing either aspartate or iso-aspartate (Robinson *et al*., 2001). To test this hypothesis two mutants were made which changed Asn–Gly pairs to Ser–Gly; ColN-T^(N4S)^ and ColN-T^(N53S)^ (Fig. 3A). ColN-T^(N4S)^ was consistently active when produced using a 16 h induction protocol while ColN-T^(N53S)^ was not. This indicates that deamidation of N4 was the cause of inactivity. N4 is just upstream of our tentative OBS1 site (8–15 aa) (Fig. 3A) and so introduction of a negatively charged aspartic/isoaspartic acid residue at this position may disrupt OmpF binding. All data presented here used T-domain produced by short induction times and a full analysis of the post-translational modifications of ColN-T will be published elsewhere.

## Discussion

### Truncation of an *E. coli* antibiotic reveals hidden antimicrobial activity of an intrinsically unfolded domain

It is a longstanding core dogma of colicin biology that the C-terminal cytotoxic domains of colicins are indispensable for bactericidal activity. Thus the retention of antibacterial toxicity when this domain is removed was entirely unexpected. Colicins are outstandingly efficient killers so even though ColN-T is four orders of magnitude less efficient than ColN, its MIC (10 μM) is still comparable to other AMPs. Furthermore, removal of both the R-domain and C-terminal cytotoxic domain results in a protein (ColN-T) which has an MIC (10 μM) still comparable to known AMPs (Melo *et al*., 2009). We have therefore shown that amino acids 1–69 define the minimal killing unit of ColN and may correlate with an original bacteriocin from which the full-length colicin developed. Acquisition of R-domain would have afforded a significant evolutionary advantage through its improved efficiency (μM to nM MIC) and finally recruitment of a dedicated pore-forming domain would generate the highly efficient three-domain ColN we know today. If this is the case, and it is notable that all colicins do possess an unstructured T-domain, the retention of antimicrobial activity in ColN-T after this time is remarkable. There is no evidence yet of this activity in other colicin IUDs, Colicin Ia T-domain is protective (Jakes and Finkelstein, 2010) while point mutations in the active sites of enzymatic colicins abolish toxicity (Garinot-Schneider *et al*., 1996)

### Loss of OBS1 can be tolerated in full-length ColN but not in ColN-T/ColN-TR

Within this relatively small IUD reside a TolA binding site and the newly identified OmpF binding site. Just like full-length ColN, both receptors were subsequently shown to be essential for ColN-T and ColN-TR toxicity. However one clear difference between ColN-T/ColN-TR and their full-length counterpart is their absolute requirement of OBS1 for activity. ColN^OBS1^ induced K^+^ efflux which was only modestly different from ColN; however, ColN-TR^OBS1^ and ColN-T^OBS1^ were completely inactive. For ColE9 in which two OBS have been identified, the presence of at least one OBS is vital for cytotoxicity (Housden *et al*., 2011). ColN, which has a much shorter T-domain than ColE9, only has one OBS and its loss can be tolerated *in vivo*. Presumably in ColN^OBS1^, which only shows a small change in MIC (supplementary data), the RP domains compensate for the loss of OmpF binding in the T-domain (Clifton *et al*., 2012). R-domain does bind to OmpF *in vitro* in the absence of detergent (Evans *et al*., 1996b; Stora *et al*., 1999) and the self-inhibition seen here implies that there is competition between the T and R domains for OmpF. It is very easy to block OBS mutants if one pre-treats the cells with ColN TolA-box mutants which cannot fully translocate. These possibly stay attached to OmpF and cannot be displaced by OBS mutants. Thus OBS binding may be a very early step in toxicity but the role that OBS plays in the physiological activity of full-length ColN and other colicins is not yet clear.

### ColN-T shares similarities with microcins and does not kill by membrane disruption

Killing bacteria by membrane disruption is very effective and small pore-forming AMPs have been suggested as new generation antibiotics. However the target of most AMPs, the lipid bilayer, is common to animals and bacteria, making selective bacterial toxicity a demanding goal. Here, ColN-T is unusual in that, although its apparent site of action is the inner membrane, it binds not lipid but specific protein targets. The release of K^+^ ions may be symptomatic of other membrane effects (Csonka and Hanson, 1991) but the rapid kinetics argue for a direct physical rather than chemical target. The size of ColN-T shares some similarities with the microcins but unlike ColN-T these generally hijack the Ton system. The 88 residue colicin V, now renamed microcin V was reported to abolish membrane potential but interestingly pore-formation could not be observed in liposomes (Yang and Konisky, 1984). Interestingly, remnants of the ColV operon exist in pAPEC-O1-ColBM, a plasmid encoding colicins B and M, hinting that ColBM-type plasmids may have evolved from ColV plasmids (Johnson *et al*., 2006).

Thus, ColN-T is the first example of an antimicrobial protein which combines a natively unfolded structure with targeted membrane permeabilization. OmpF and TolA homologues are widely found in Gram-negative bacterial pathogens making this killing mechanism a tangible target for the development of small molecules with low MICs suitable for antibacterial drug therapy (Strom *et al*., 2003; Gillor *et al*., 2004).

### ColN-TR self-inhibition suggests ColN may hijack two OmpF molecules in the outer membrane

Colicin self-inhibition has been demonstrated before by Jakes and Finkelstein (2010). When performing the spot test assay using high concentrations of ColIa they observed zones of inhibition with a clear periphery but turbid centres. Furthermore, the same self-inhibition was detected in liquid culture, with ColIa found to kill more efficiently at ∼ 170 mpc than at 8700 mpc. The basis of self-inhibition is that a single ColIa molecule binds two separate Cir proteins (∼ 5000 copies per cell) (Konisky and Cowell, 1972) via its R- and T-domains respectively. Self-inhibition at high concentrations is due to binding of the high-affinity R-domain to every copy of Cir on the cell which prevents the essential binding of the lower affinity T-domain. ColN-TR self-inhibition occurs at multiplicities exceeding 10^5^ mpc suggesting that the R-domain of ColN-TR binds OmpF which is present at ∼ 10^5^ copies per cell and inhibits T-domain killing (Sodergren *et al*., 1985). Jakes and Finkelstein (2010) demonstrated self-inhibition using full-length ColIa, but we see no self-inhibition with full-length ColN and only with Col-TR. Thus self-inhibition implies that both colicins Ia and N use two copies of their Omp receptor. In the case of ColN one interaction, OBS, can be deleted and it is interesting that Colicin S4 has two identical receptor (OmpW)-binding domains of which only one needs to be functional for toxicity (Arnold *et al*., 2009).

## Experimental procedures

### Bacterial strains

The standard strain used for assaying colicin killing is BE3000 (*OmpC*^−^). To check for OmpF and TolA dependence of colicin activity the strains BZB1107 (*OmpC*^−^*, OmpF*^−^*, LamB*^−^) and JC207 (*TolA*^−^) were used respectively. To screen for TolQ and R dependence of colicin activity we used the Keio parental strain BW25113 [*rrnB3* Δ*lacZ4787 hsdR514* Δ(*araBAD*)*567* Δ(*rhaBAD*)*568*], Δ*tolQ* (*tolQ786::kan*) and Δ*tolR* (*tolR787::kan*).

### Constructs used in this work

DNA sequences encoding the constructs used in this work were synthesized by GeneArt (Regensburg, Germany). In addition to encoding the colicin constructs in the forward frame the cognate colicin N immunity protein was encoded in the reverse frame. All GeneArt DNA sequences were synthesized with a C-terminal –SSHHHHHH tag with NdeI (5′) and BamHI (3′) restriction sites for subcloning into pET3a (Novagen).

### Protein purification

OmpF was purified as described previously (Lakey *et al*., 1985), precipitated in cold ethanol and resuspended in 20 mm sodium phosphate, pH 7.2, 300 mM NaCl, 1% (v/v) octyl-polyoxyethylene (POE) (Enzo). EDTA was then added to the OmpF solution to a final concentration of 10 mM before loading (0.5 mg ml^−1^) onto a size exclusion column (Superose 12) equilibrated 20 mm sodium phosphate buffer, pH 7.2, 300 mM NaCl, 10 mM EDTA, 1% (v/v) octyl-polyoxyethylene (POE) (Enzo). All colicin constructs were purified as described previously (Fridd *et al*., 2002), followed by dialysis into 50 mm sodium phosphate, pH 7.6, 300 mm NaCl.

### Spot test assay

Colicin domain mutants were assayed for activity using the established spot test dilution assay (Pugsley and Schnaitman, 1978).

### K^+^ efflux assay using live cells

A single colony of *E. coli* BE3000 cells was used to inoculate 5 ml of 1× LB and grown overnight, shaking at 37°C. After 16 h growth 1 ml of cells was used to inoculate 100 ml of 1× LB supplemented with 10 mM KCL. Cells were grown by shaking at 37°C, until an OD_600_ of 0.5–0.6. Cells were harvested by centrifugation at 3000 *g* at 25°C for 15 min. The supernatant was carefully discarded the cell pellet washed with 4× 1 ml aliquots of 100 mM sodium phosphate, pH 7.0 (assay buffer) before resuspension in 1 ml of 100 mM sodium phosphate, pH 7.0, 5% glycerol (loaded assay buffer). Cells were stored on ice and used within 3 h of preparation after allowing for 30 min equilibration on ice. Following equilibration a defined aliquot containing 5 × 10^9^ cells was taken and added to a jacketed K^+^ efflux vessel maintained stirring at 37°C containing 6 ml of pre-warmed assay buffer. Data recording was started upon addition of cells using an ion-selective K^+^ electrode, double junction lithium acetate reference electrode and a temperature probe. Upon addition to the vessel the cells re-accumulate K^+^ over approximately 5 min until a stable baseline is reached. Unless otherwise stated colicin was added to the vessel 60 s after the cells had reached a stable baseline. Data were normalized to zero at the point of colicin addition and measurements continued for 7 min, with data points being taken every 5 s. For all K^+^ efflux measurements the basal rate of K^+^ loss from 5 × 10^9^ cells was recorded to serve as a negative control. The results shown are representative of a minimum of three independent experiments.

### MICs

A single colony of *E. coli* BE3000 cells was added to 5 ml of 1× LB and grown overnight, shaking at 37°C. After 16 h growth 1 ml of cells was used to inoculate 50 ml of 1× LB. Cells were grown by shaking at 37°C, until an OD_600_ of 1.5. An aliquot of cells was then diluted 1/100 with 1× LB and 5 μl of diluted cells added to each well of a 96-well plate. Stocks of 100 μM and 1 nm of ColN domain mutants were prepared in 1× LB and each of the stock solutions serially diluted to produce a dilution series of 10 μM, 1 μM, 10 nM, 1 nM, 0.1 nM, 10 pM and 1 pM. MIC was scored as the lowest concentration of a particular colicin domain mutant at which no growth of the *E. coli* was observed after 18 h of incubation while shaking at 37°C.

### Preparation of K^+^ loaded liposomes

Fifty milligrams of *E. coli* natural lipid extract (Avanti) (57.5% PE, 15.1% PG, 9.8% CA, 17.6% unknown) in chloroform was dried and stored under vacuum for 3 h to remove remaining solvent. Lipids were then resuspended in 5 ml of 100 mM potassium phosphate pH 7.0 to give a 10 mg ml^−1^ stock. The solution was then sonicated on ice for 20 min before centrifugation at 13 000 *g* for 30 min. The supernatant containing the liposomes was exchanged for K^+^-free 100 mM sodium Phosphate pH 7.0 using a PD10 column.

### Liposome efflux assay

ColN-T or melittin (positive control) were added 60 s after liposome addition. Additional runs were carried out where liposomes alone (negative control) were added to the vessel to assess basal K^+^ leakage from the vesicles. Data were normalized to zero at the point of liposome addition and measurements typically continued for ∼ 15 min, with data points being taken every 5 s.

### Tryptophan fluorescence

Tryptophan fluorescence emission spectra were measured with a Varian spectrofluorimeter (Varian, USA) using a 280 nm excitation wavelength. Slits were set at 5 nm for excitation and emission. Emission spectra were recorded between 300 and 450 nm with a scan rate of 600 nm min^−1^ in a 0.5 cm pathlength cuvette at 25°C. Spectra in the presence of liposomes were corrected for light scattering by subtracting the corresponding liposome background. Under each condition four spectra were averaged.

### Circular dichroism

Circular dichroism was measured using a J-810 spectropolarimeter (Jasco, Japan) and Quartz-Suprasil circular cell (Hellma, GmbH & Co., Germany). The far-UV spectrum was recorded over 10 accumulations between 190 and 260 nm before averaging the data. Processing of the spectra included subtracting the buffer spectrum from the sample spectrum before conversion into standard units of Δε (M^−1^ cm^−1^).

### Isothermal titration calorimetry (ITC)

ITC measurements were performed using a MicroCal ITC_200_ thermostatted at 25°C, with all protein samples prepared in 20 mM K^+^ phosphate, pH 7.5, 150 mM NaCl, 1% Octyl-poe. OmpF was present in the sample cell at a concentration of 40–50 μM per monomer with the colicin concentration in the syringe varying from 400 to 700 μM. Binding isotherms were analysed using the manufacturer's software.
