# Supplementary material for: The unstructured domain of colicin N kills Escherichia coli
Source: Mol Microbiol. 2013 Jun 5;89(1):84–95. doi: 10.1111/mmi.12260 (PMC3739937; doi:10.1111/mmi.12260)
Supplement: Supplementary file 1 [file mmi0089-0084-SD1.pdf]

Supplementary online information for:

The intrinsically unstructured translocation domain of colicin N kills *Escherichia coli.*

*Christopher L. Johnson[1], Helen Ridley[1], Robert J. Pengelly[1] and Jeremy H. Lakey[1]**

1 Centre for Bacterial Cell Biology, Institute for Cell and Molecular Biosciences, Faculty of Medical Sciences, University of Newcastle, Framlington Place, Newcastle- upon-Tyne, NE2 4HH, UK.

* E-mail: jeremy.lakey@ncl.ac.uk

Supplementary figures.

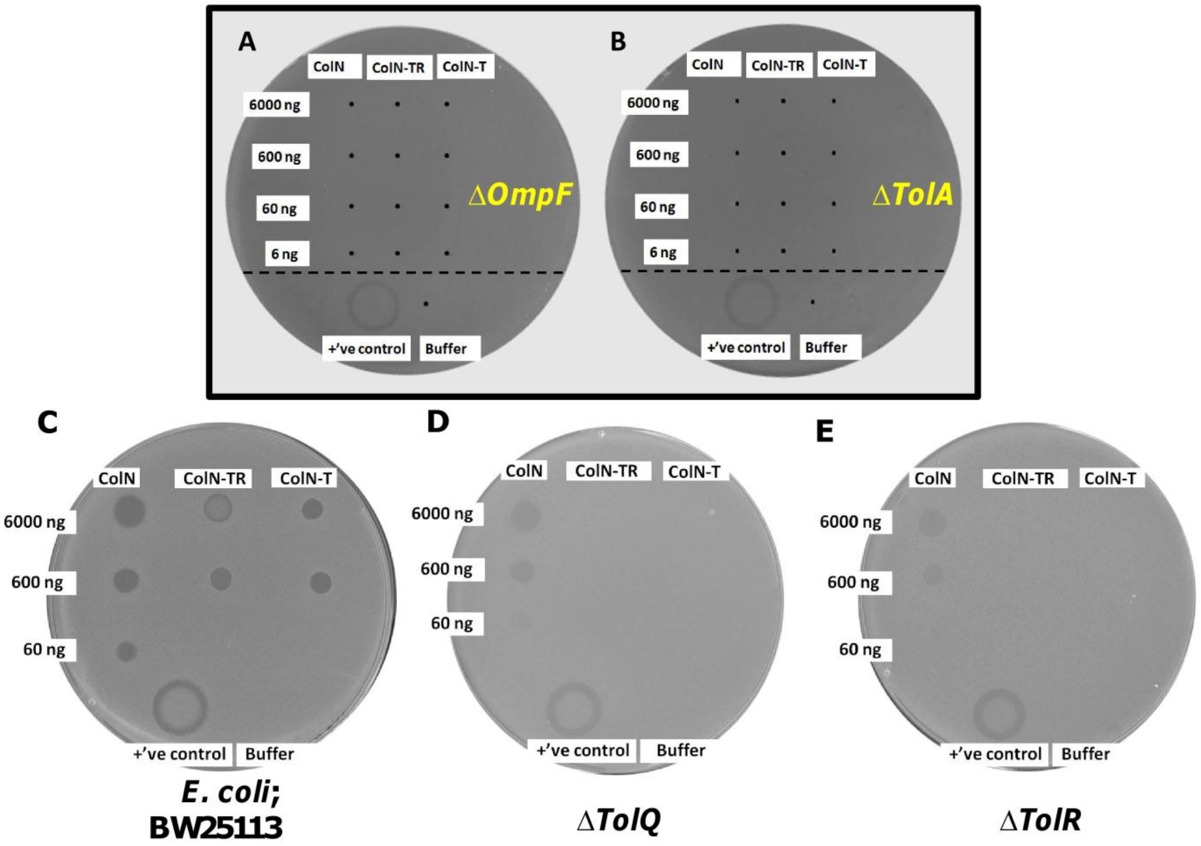

Figure S1 (A&B). Spot test assay to demonstrate the OmpF, TolAQR dependency of ColN-TR and ColN-T killing. 2µl volumes corresponding to 6000 ng, 600 ng, 60 ng and 6 ng of ColN, ColN-TR and ColN-T were spotted onto a lawn of *E. coli*, either lacking **A**; OmpF (BZB1107) , **B**; TolA (JC207), **D;** TolQ (*E. coli*; BW25113Δ*tolQ*), **E;** TolR (*E. coli*; BW25113Δ*tolR*). The control strain *E. coli*; BW25113 is shown in (**C**) .As a negative control 2 µl of the buffer in which the colicins were diluted (50 mM sodium phosphate, pH 7.5, 300 mM NaCl) was spotted. As a positive control 2µl (3000 ng) of ColIa (Cir and TonB dependent) was spotted.

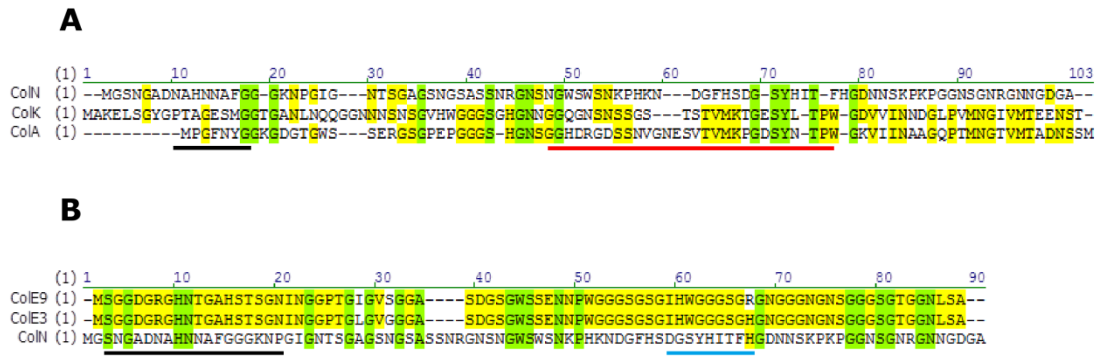

Figure S2

Figure S2. Multiple alignments of colicin T-domains. (**A**) Multiple alignment of the pore forming colicins ; colicin N (ColN), colicin K (ColK) and colicin A (ColA) which require OmpF for import and contain a TolA box. The minimal ColN OBS1 site is underlined in black and the ColN TolA box is underlined in red. (**B**). Multiple alignment of colicin N (ColN) with the enzymatic colicins E3 (ColE3) and E9 (ColE9) which require OmpF for import. The ColE9 OBS1 site is underlined in black and the OBS2 site in blue.

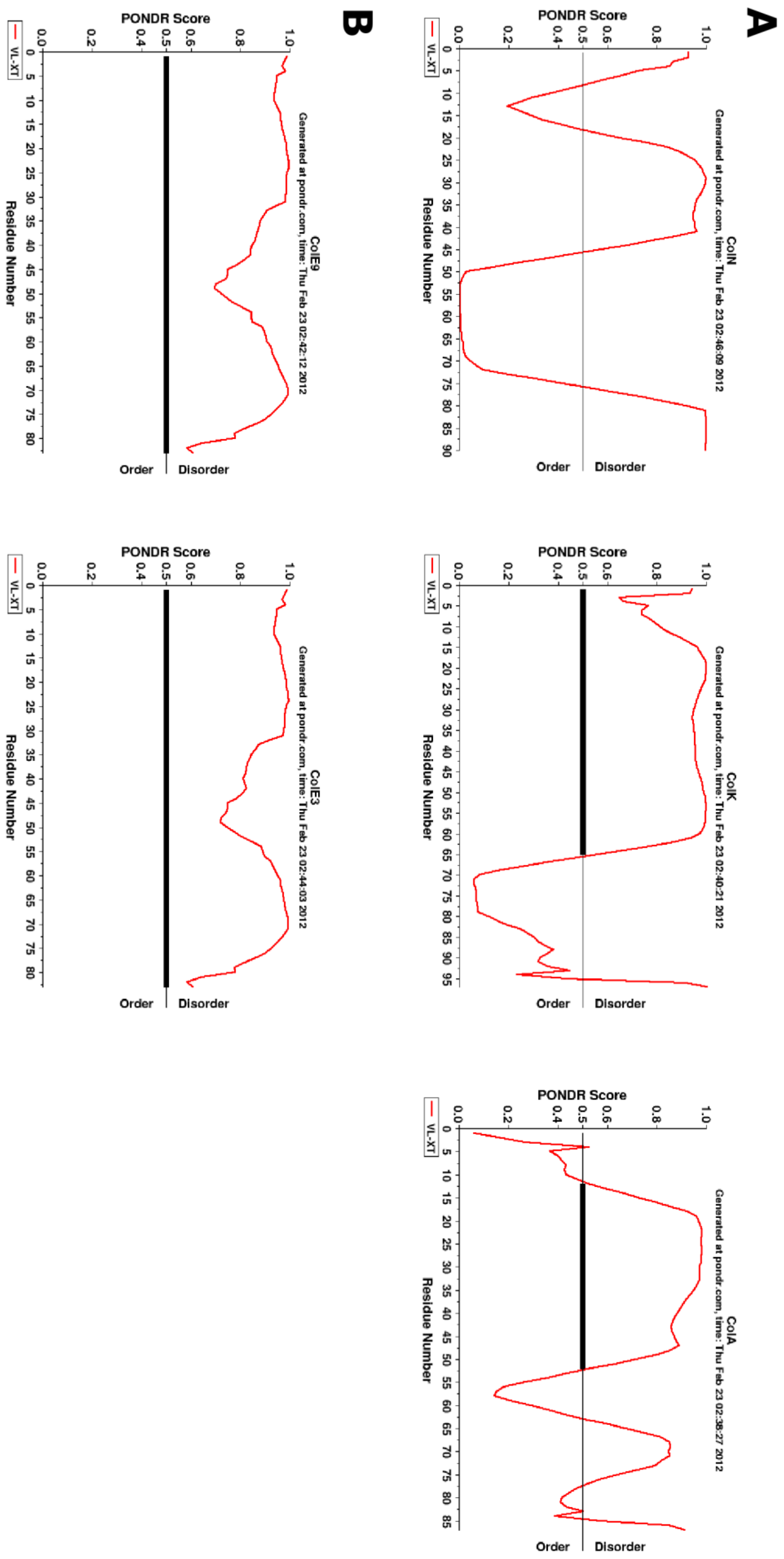

Figure S3. PONDR plots of colicins which utilise OmpF for import. (A) PONDR plots of the pore forming colicins; colicin N (ColN), colicin K (ColK) and colicin A (ColA). (B) PONDR plots of the enzymatic colicins; colicin E9 (ColE9) and colicin E3 (ColE3).

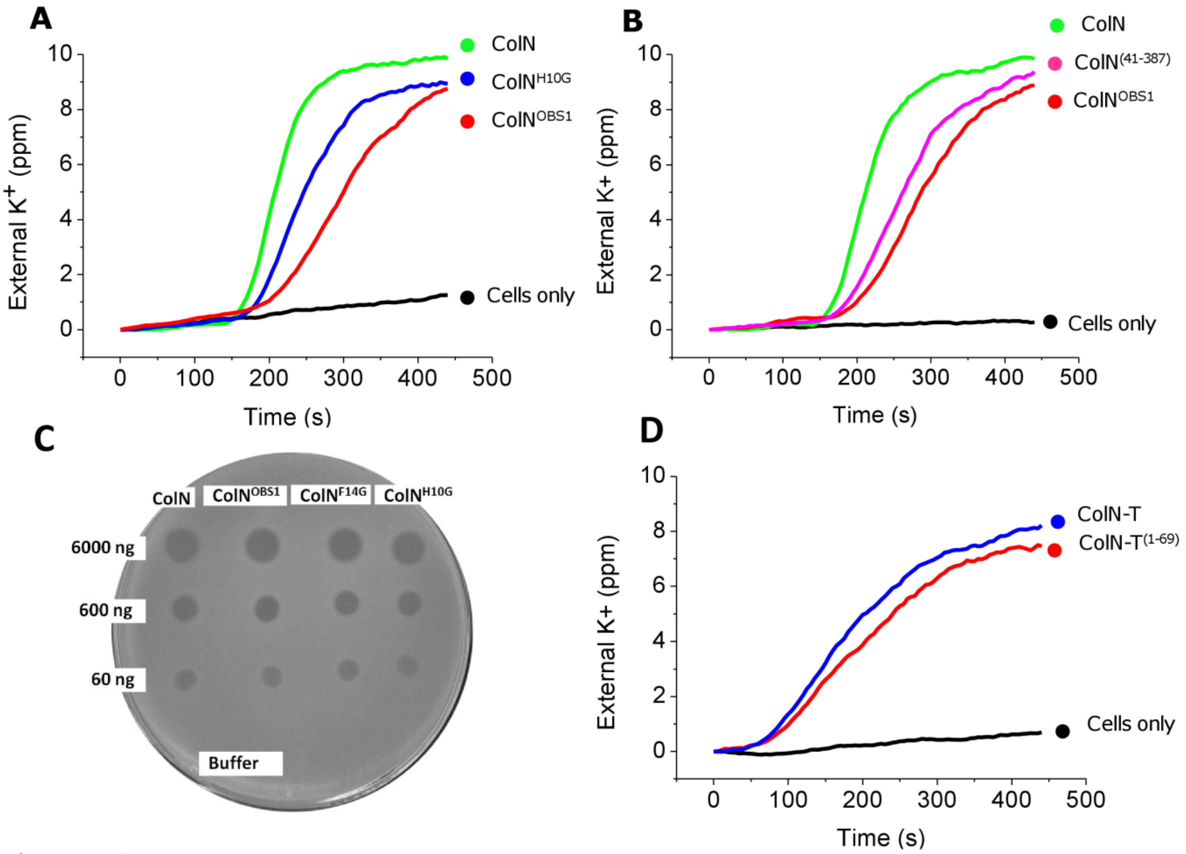

Figure S4 A direct comparison of ColN OBS mutants. K^+ efflux (A) ColN^{OBS1} (red) and ColN^{H10G} (blue) were added at 600 mpc. ColN (green) added at 600 mpc is shown for reference, negative control (black). All colicins were added 120 seconds. (**B**) ColN^{OBS1} (red) and ColN$^{(41\text{-}387)}$ (magenta) were added at 600 mpc. ColN (green) added at 600 mpc is shown for reference, negative to which no colicin is added (black). All colicins were added 120 seconds. **(C) Spot tests** 2µl volumes corresponding to 6000 ng, 600 ng, and 60 ng of ColN, ColN^{OBS1}, ColN^{F14G} and ColN^{H10G} were spotted onto a lawn of BE3000. As a negative control 2 µl of the buffer in which the colicins were diluted (50 mM sodium phosphate, pH 7.5, 300 mM NaCl) was spotted. (**D) K^+ efflux of Colicin N-T $^{(1\text{-}69)}$.** ColN-T (blue) and ColN-T$^{(1\text{-}69)}$ (red) were added at 6 x 10^5 MPC at 0 seconds. Negative control to which no colicin is added to cells (black).

Supplementary Table

Table S1. MIC values of ColN OBS1 mutants.

**Colicin Construct**	**MIC**
$ColN^{OBS1}$	10 nM
$ColN^{F14G}$	10 nM
$ColN^{H10G}$	1 nM
$ColN\text{-}TR^{OBS1}$	n/a
