# Supplementary figures and images for: The unstructured domain of colicin N kills Escherichia coli
Source: Mol Microbiol. 2013 Jun 5;89(1):84–95. doi: 10.1111/mmi.12260 (PMC3739937; doi:10.1111/mmi.12260)

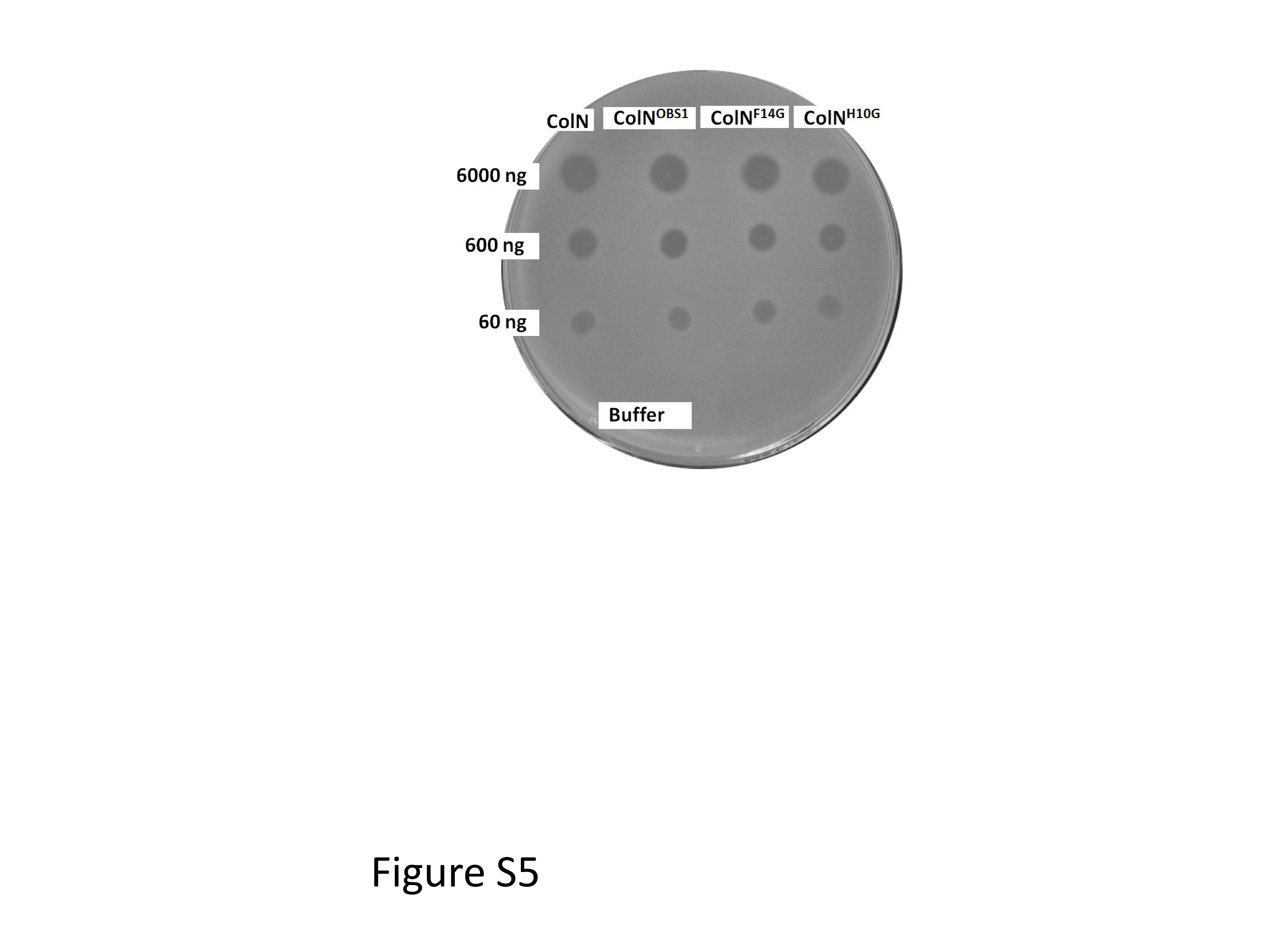

Supplement: Supplementary file 2 [file mmi0089-0084-SD2.tif]
